# *Lactobacillus johnsonii* JNU3402 Ameliorates Age‐Related Liver Dysfunction Through Stimulating PGC‐1α‐Mediated SIRT1 Expression

**DOI:** 10.1002/biof.70069

**Published:** 2025-12-04

**Authors:** Eunjeong Hong, Garam Yang, Sejong Oh, Eungseok Kim

**Affiliations:** ^1^ Department of Biological Sciences College of Natural Sciences, Chonnam National University Gwangju Republic of Korea; ^2^ Division of Animal Science College of Agriculture & Life Sciences, Chonnam National University Gwangju Republic of Korea

**Keywords:** aging, *Lactobacillus johnsonii* JNU3402, metabolic dysfunction‐associated steatotic liver disease, mitochondrial function, senescence

## Abstract

*Lactobacillus johnsonii* JNU3402 (LJ3402) has previously been reported to ameliorate diet‐induced hepatic steatosis. Because aging is tightly linked to metabolic disease, we hypothesized that LJ3402 might protect against age‐related metabolic abnormalities in the liver. This study presents data demonstrating that LJ3402 administration reduces hepatic dysfunction in 24‐month‐old mice alongside the alleviation of general aging phenotypes. Furthermore, LJ3402 increased hepatic expression of genes involved in mitochondrial function and decreased senescence markers, thereby limiting age‐related mitochondrial dysfunction and hepatocyte senescence, contributing to the attenuation of metabolic dysfunction‐associated steatotic liver disease (MASLD) progression. Mechanistically, LJ3402 enhanced sirtuin 1 (SIRT1) expression in AML12 hepatocytes by stimulating the peroxisome proliferator‐activated receptor gamma coactivator 1‐alpha (PGC‐1α) coactivation of peroxisome proliferator‐activated receptor alpha (PPARα). Consequently, SIRT1 suppressed p53 acetylation and activity in senescent AML12 cells, reducing senescence markers and mitochondrial dysfunction. Thus, LJ3402 suppresses mitochondrial dysfunction and senescence of hepatocytes by stimulating the PGC‐1α–SIRT1–p53 pathway, reducing age‐related hepatic lipid accumulation.

## Graphical abstract

LJ3402 improves mitochondrial function and suppresses cellular senescence in hepatocytes by stimulating the PGC‐1α–SIRT1–p53 pathway, alleviating age‐related liver dysfunction.

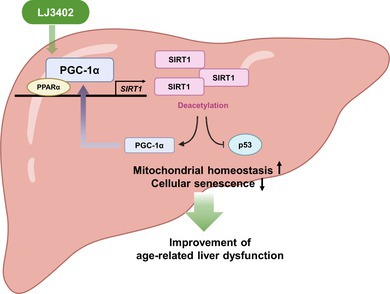

**Abbreviations**
ACC: acetyl‐CoA carboxylase
ALT: alanine aminotransferase
AML12: alpha mouse liver 12
AST: aspartate aminotransferase
CAT: catalase
ChIP: chromatin immunoprecipitation
CM: conditioned medium
CS: citrate synthase
CTP: carnitine palmitoyl transferase
FFA: free fatty acid
GTT: glucose tolerance test
ITT: insulin tolerance test
MASLD: metabolic dysfunction‐associated steatotic liver disease
NRF: nuclear factor erythroid 2–related factor
OCR: oxygen consumption rate
PFTα: pifithrin‐α
PGC‐1α: peroxisome proliferator‐activated receptor gamma coactivator 1‐alpha
PPAR: peroxisome proliferator‐activated receptor
SASP: senescence‐associated secretory phenotype
SA‐β‐gal: senescence‐associated β‐galactosidase
SIRT1: sirtuin 1
SOD: superoxide dismutase
SREBP‐1c: sterol regulatory element–binding protein 1c
TFAM: mitochondrial transcription factor A
TG: triglyceride

## 1 Introduction

Aging is a process of gradual decline in physical and physiological functions, which increases susceptibility to various age‐related diseases, such as metabolic diseases [1]. Meanwhile, the liver is a central organ responsible for maintaining systemic energy homeostasis by orchestrating glucose and lipid metabolism in response to energy demands [2]. With age, hepatic function deteriorates, contributing to the higher prevalence of fatty liver disease in older individuals [3].

Mitochondria are dynamic organelles that serve as key regulators of cellular energy homeostasis and stress responses. However, aging promotes mitochondrial dysfunction in hepatocytes, leading to reduced oxidative phosphorylation and increased production of reactive oxygen species (ROS), thereby promoting excessive lipid accumulation in the liver [1, 3, 4]. Mitochondrial dysfunction is closely linked to cellular senescence, a state of irreversible cell cycle arrest, during which senescent cells increase the secretion of proinflammatory cytokines, chemokines, growth factors, and matrix‐degrading factors, collectively termed the senescence‐associated secretory phenotype (SASP) [5, 6]. Accumulating senescent cells remodel the tissue microenvironment via SASP factors, propagating cellular senescence to neighboring cells with age [7]. Recent studies directly connect hepatocyte senescence with hepatic lipid accumulation, implicating mitochondrial dysfunction as a key driver [6, 8]. Conversely, removing senescent cells alleviates hepatic steatosis [9], underscoring the importance of preserving cellular integrity and mitochondrial function to prevent age‐related fatty liver disease.

Sirtuin 1 (SIRT1), an NAD^+^‐dependent deacetylase, functions as a metabolic and redox sensor under metabolically stressful conditions, preventing metabolic abnormalities by modulating various metabolic transcription regulators [10, 11]. Peroxisome proliferator‐activated receptor gamma coactivator 1‐alpha (PGC‐1α) drives mitochondrial biogenesis and energy expenditure by functioning as a coactivator of transcription factors such as nuclear factor erythroid‐2–related factor 2 (Nrf2) and peroxisome proliferator‐activated receptors (PPARs) [12, 13, 14]. SIRT1 directly deacetylates and activates PGC‐1α, thereby maintaining mitochondrial homeostasis [10, 15]. In addition SIRT1 suppresses cellular senescence by regulating p53 [10]. Notably, p53 can downregulate PGC‐1α under stressors including DNA damage, telomere attrition, and cancer, thereby contributing to mitochondrial dysfunction and senescence [16, 17, 18]. Although the SIRT1–PGC‐1α pathway is established in metabolic homeostasis, whether PGC‐1α regulates SIRT1 transcription remains unexplored, particularly in relation to age‐related liver pathophysiology.

Probiotics are live microorganisms that confer health benefits to the host by regulating metabolic and immune pathways when administered in adequate amounts. Meanwhile, accumulating evidence indicates that select strains attenuate metabolic disorders, including diabetes and fatty liver disease [19, 20, 21, 22]. We previously showed that *Lactobacillus johnsonii* JNU3402 (LJ3402) prevents metabolic dysfunction‐associated steatotic liver disease (MASLD) by limiting hepatic lipid accumulation in diet‐induced obese male mice [23]. Because MASLD is more prevalent in men, we focused on male mice to assess whether LJ3402 mitigates age‐related hepatic dysfunction. Here, we demonstrate that LJ3402 improves mitochondrial function and inhibits hepatocyte senescence through the PGC‐1α–SIRT1–p53 pathway.

## 2 Experimental Procedures

### 2.1 Animal Experimentation

Male C57BL/6J mice (Central Animal Laboratory, Daejeon, Korea) were fed a normal diet (ND; 16% of total calories from fat; Lab Diet, St. Louis, MO, USA) under a 12 h light/dark cycle. LJ3402 cells were cultured in MRS broth (BD, Difco Laboratories, Detroit, MI, USA) under anaerobic conditions at 37°C for 24 h and harvested by centrifugation at 4000*g* for 5 min. Pellets were resuspended in sterile PBS to 1.0 × 10^8^ CFU/mL. Mice received 200 μL by oral gavage daily. LJ3402 dosing suspensions were prepared fresh immediately before administration. Ten‐month‐old mice were gavaged with 200 μL PBS or LJ3402 suspension for 14 months (*n* = 8 per group). The young mice group comprised 2‐month‐old C57BL/6J male mice fed the ND (*n* = 8). All mice remained in good health and were euthanized with CO_2_ at specified time points. Blood was collected into heparinized tubes, centrifuged at 1000*g* for 15 min at 4°C to separate plasma, and stored at −80°C. Liver tissue was snap‐frozen in liquid nitrogen and stored at −80°C until use. All animal experiments were approved by the Institutional Animal Care and Use Committee of Chonnam National University (approval number: CNU‐IACUC‐YB‐R‐2022‐26).

### 2.2 Histochemistry Staining

Liver tissue was isolated from each group of mice and fixed with 10% formalin in PBS before being embedded in FCS 22 frozen section media (Leica Biological System, Richmond, IL, USA). Embedded samples were cryosectioned at 7 μm using a cryostat (CM1950, Leica Biosystem, Nussloch, Germany). Sections were stained with hematoxylin–eosin, Oil Red O, and senescence‐associated β‐galactosidase, then imaged by bright field on a BioTek Cytation 5 cell‐imaging multimode reader (BioTek, Winooski, VT, USA).

### 2.3 Plasma and Tissue Analyses

Tissue triglyceride (TG) and free fatty acid (FFA) contents and plasma TNFα, IL‐1β, aspartate aminotransferase (AST), and alanine aminotransferase (ALT) were measured using the following kits: TG Quantification and FFA Quantification (SCG Biomax, Seoul, Korea); AST Activity and ALT Activity (Abcam, Cambridge, MA, USA); TNFα ELISA (Invitrogen, Carlsbad, CA, USA); and IL‐1β ELISA (R&D Systems, Minneapolis, MN, USA), according to the manufacturers' protocols. After 14 h of fasting, a glucose tolerance test (GTT) was performed by intraperitoneal injection of glucose (1.5 g/kg body weight). For the insulin tolerance test (ITT), mice were fasted for 4 h and then injected intraperitoneally with insulin (0.75 U/kg body weight). Blood glucose was measured by tail bleeding at 0, 15, 30, 45, 60, and 90 min after injection using a CERA‐CHEK 1 Code meter (model G400; Green Cross Medis Corp., Yong in, Korea). The area under the curve (AUC) for GTT or ITT was calculated using the trapezoidal rule, which estimates the total area by integrating the trapezoids formed by glucose concentration values plotted over time.

### 2.4 Plasmids and Transfection

The pGL2–human SIRT1 promoter‐luciferase plasmid was provided by Dr. Man‐Wook Hur (Yonsei University, Seoul, Korea). The pBluescript II KS(+)‐p21 promoter‐luc plasmid was purchased from Addgene (MA, USA) and designated as p21 promoter‐luc. The pcDNA‐flag–SIRT1, pcDNA3–PGC‐1α, and pcDNA3–p53 plasmids have been described previously [19, 24]. Small interfering RNA (siRNA) targeting mouse SIRT1 (SIRT1 siRNA) or PGC‐1α (PGC‐1α siRNA) was purchased from Bioneer (Daejeon, Korea). Cells were transfected with the appropriate plasmids using the Lipofectamine 3000 transfection kit (Thermo Fisher Scientific, Cleveland, OH, USA) according to the manufacturer's instructions.

### 2.5 Cell Culture and LJ3402‐Conditioned Medium (LJ3402‐CM)

HEK293T cells were cultured in Dulbecco's modified Eagle's medium (DMEM) with 5% fetal bovine serum (FBS) and 1% antibiotics. Alpha mouse liver 12 (AML12) cells were cultured in DMEM/F‐12 containing 10% FBS, 1% insulin–transferrin–selenium–pyruvate supplement, 0.1 μM dexamethasone, and 1% antibiotics. To induce senescence in AML12 hepatocytes, cells were treated with 1 mM H_2_O_2_ for 1 h on day 1 and 750 μM H_2_O_2_ for 1 h on days 2–7 [25]. LJ3402‐conditioned medium (LJ3402‐CM) was prepared as described previously and administered to cells at 1/100 of the culture‐medium volume [23].

### 2.6 Reverse Transcription–Quantitative PCR (RT‐qPCR), chromatin Immunoprecipitation (ChIP), immunoblotting, and Immunoprecipitation (IP)

Total RNA extraction and reverse transcription were performed as described previously [20]. RT‐qPCR was performed with the TOPreal SYBR Green qPCR kit (Enzynomics, Daejeon, Korea), and *36B4* mRNA was used as the internal control for gene quantification. ChIP assays were performed in AML12 cells using anti‐PPARα and anti‐PGC‐1α antibodies (Santa Cruz, CA, USA). The primer sequences used for RT‐qPCR are described in Table 1. Immunoblotting used anti‐p21, anti‐PGC‐1α, anti‐β‐actin (Santa Cruz), anti‐p53, anti‐γH2AX, anti‐acetylated lysine (Cell Signaling), and anti‐SIRT1 (Abcam) antibodies. For acetylation assays, anti‐PGC‐1α and anti‐p53 antibodies were used for immunoprecipitation, and immunoprecipitates were analyzed by immunoblotting using anti‐acetylated lysine antibody.

**TABLE 1:** Primers used for RT‐qPCR.

Gene	Gene primer sequences	
	Forward (5′–3′)	Reverse (5′–3′)
*p21*	AGTGCAAGACAGCGACAA	CGAGAACGGTGGAACTTTGAC
*p16*	GAACTCTTTCGGTCGTACCC	TGGGCGTGCTTGAGCTGA
*p53*	CCACCACACTATGTCGAAAAGT	ATGGCCATCTACAAGCAGTC
*SIRT1*	AGTTCCAGCCGTCTCTGTGT	CTCCACGAACAGCTTCACAA
*Pgc1a*	GAGACTTTGGAGGCCAGCA	CGCCATCCCTTAGTTCACTGG
*Nrf1*	ATGACTCGGACATCCTCAAC	TGTCTGCTGTCTCTTTCGG
*Tfam*	AATTGCAGCCCTGTGGAG	TCAGCTGACTTGGAGTTAGC
*Sod*	TCTAAGAAACATGGTGGCCC	TCCCAGCATTTCCAGTCTTT
*Cat*	CCGGCACATGAATGGCTA	AACGTCCAGGACGGGTAATT
*36B4*	AGATGCAGCAGATCCGCAT	ATATGAGGCAGCAGTTTCTCCAG
*D‐loop*	AATCTACCATCCTCCGTG	GACTAATGATTCTTCACCGT
*Gapdh*	GTTGTCTCCTGCGACTTCA	GGTGGTCCAGGGTTTCTTA

ChIP primer	Gene primer sequences	
	Forward (5′–3′)	Reverse (5′–3′)
*PPRE (SIRT1 promoter)*	GTATTTTTATGCTGGGCTTGC	AACTCTGTAGATCAGGCTGTC

### 2.7 Mitochondrial DNA Copy Number, Enzymatic Assays, Cellular ROS, and Oxygen Consumption Rate (OCR) Analyses

DNA extraction and mitochondrial DNA (mtDNA) copy number analysis were performed in AML12 hepatocytes as described previously. Primer sequences for mtDNA copy number analysis are listed in Table 1. Citrate synthase (CS), catalase (CAT), and superoxide dismutase (SOD) activities in AML12 hepatocytes were measured with the corresponding activity assay kits (SCG Biomax, Seoul, Korea) according to the manufacturer's protocols. Cellular ROS levels were measured using the DCFDA/H2DCFDA Cellular ROS Assay Kit (Abcam). Oxygen consumption rate (OCR) in AML12 hepatocytes was measured under basal conditions and after sequential additions of 2.5 μM oligomycin, 1 μM FCCP, and 1 μM rotenone/antimycin A using the Seahorse Bioscience XFp analyzer, according to the manufacturer's instructions. For normalization, nuclei were stained with Hoechst 33342 (Thermo Fisher Scientific, Cleveland, OH, USA) and counted using a BioTek Cytation 5 cell imaging multimode reader (BioTek, Winooski, VT, USA).

### 2.8 Statistical Analysis

All data are presented as mean ± SEM from three or more independent biological experiments. Statistical analyses were performed in GraphPad Prism using two‐way ANOVA (analysis of variance) with Tukey's multiple comparisons test. *p* < 0.05 was considered statistically significant.

## 3 Results

### 3.1 LJ3402 Administration Ameliorates General Aging Phenotypes

**FIGURE 1:**
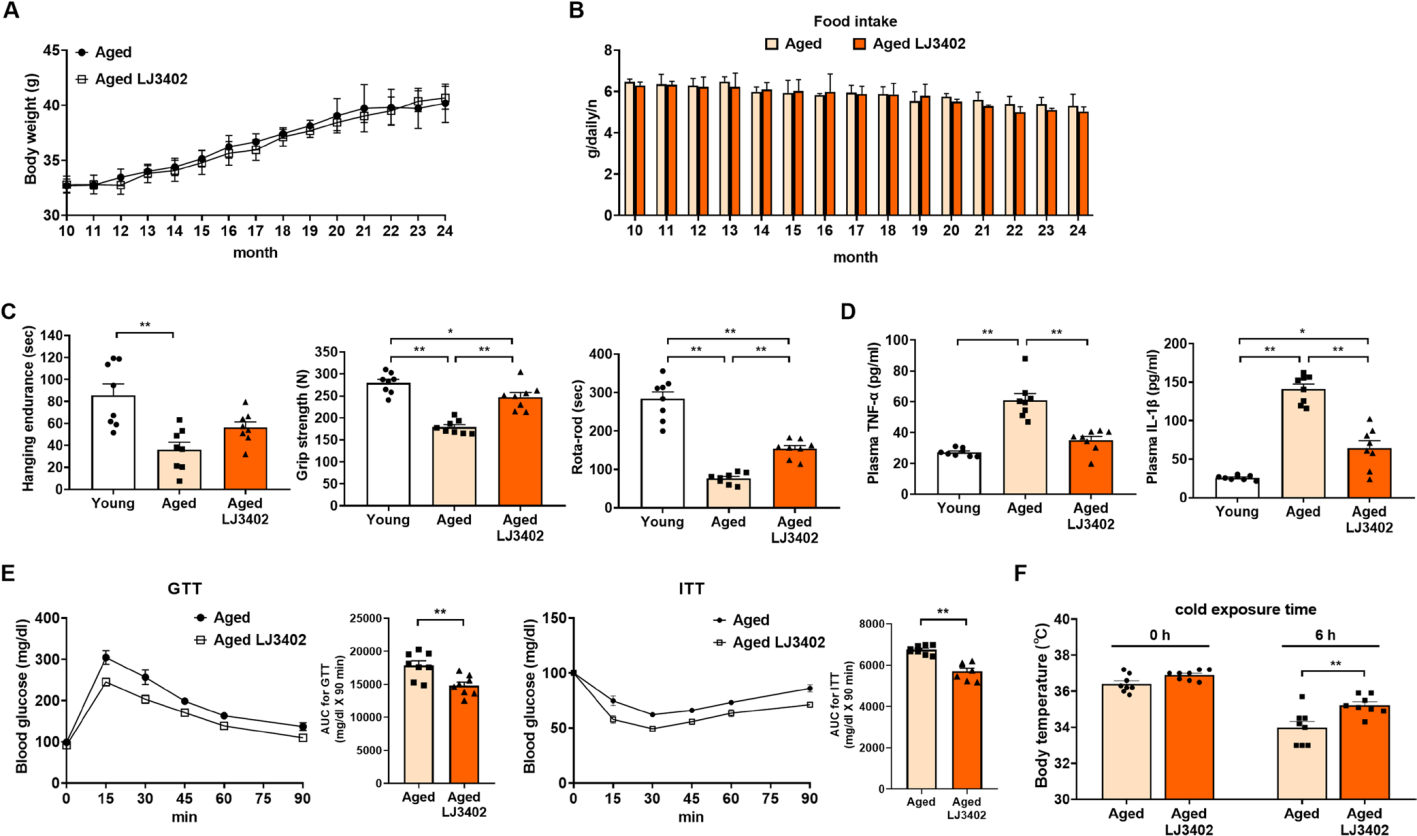
The effect of *Lactobacillus johnsonii* JNU3402 (LJ3402) administration on age‐associated phenotypes in mice. Ten‐month‐old C57BL/6J male mice were fed a normal diet (ND) without or with oral supplementation of LJ3402 for 14 months (*n* = 8 per group) and designated as aged and aged‐LJ3402 mice, respectively. Two‐month‐old ND‐fed C57BL/6J male mice were designated as the young group. Body weight gain (A) and food intake (B) of mice during aging. Plasma levels of TNF‐α and IL‐1β (C), and physical performance (hanging endurance, grip strength, rota‐rod) tests (D) in young and aged mice. (E) Glucose tolerance test (GTT) and insulin tolerance test (ITT) in 24‐month‐old mice. The AUC for the GTT and ITT data is shown on the right. (F) Rectal temperatures of mice at room temperature (0 h) and after cold exposure (4°C for 6 h). All data are presented as the mean ± standard error value (SEM). * *p* < 0.05 and **p < 0.01.

Ten‐month‐old male C57BL/6J mice were fed a chow diet with oral administration of LJ3402 or vehicle (PBS) for 14 months (hereafter referred to as “aged‐LJ3402 mice” and “aged‐control mice”, respectively). At 24 months, aged‐LJ3402 mice exhibited slightly higher body weight than aged‐control mice; however, the difference did not reach statistical significance (Figure 1A). Similarly, food intake did not differ between groups throughout the study (Figure 1B). To determine whether LJ3402 administration improves age‐related physical decline, we measured maximal walking speed, muscle strength, and physical endurance using Rota‐Rod, grip strength, and hanging endurance tests. As expected, aged‐control mice showed 36%–67% lower physical performance than 2‐month‐old mice (young mice) (*p* < 0.05; Figure 1C). In contrast, aged‐LJ3402 mice had 1.38–1.53‐fold higher Rota‐Rod performance, grip strength, and hanging endurance than aged‐control mice (*p* < 0.05). In addition, plasma TNFα and IL‐1β were 1.9‐fold and 4.7‐fold higher, respectively, in aged‐control than in young mice, whereas LJ3402 administration reduced these cytokines by 30%–47% in aged mice (*p* < 0.05; Figure 1D), indicating that LJ3402 administration alleviates physical decline and systemic inflammation. Because aging is closely associated with metabolic abnormalities, we next examined insulin sensitivity. In GTT and ITT, aged‐LJ3402 mice consistently had lower plasma glucose than aged‐control mice at all post‐injection time points (*p* < 0.01; Figure 1E). Moreover, core body temperature was slightly higher at 25°C in aged‐LJ3402 mice than in aged‐control mice but not significantly different. After 6 h of cold exposure at 4°C, aged‐LJ3402 mice maintained significantly higher core body temperature than aged‐control mice (*p* < 0.01; Figure 1F). These results indicate that LJ3402 administration mitigates general hallmarks of aging.

### 3.2 LJ3402 Attenuates Age‐Related Liver Dysfunction

**FIGURE 2:**
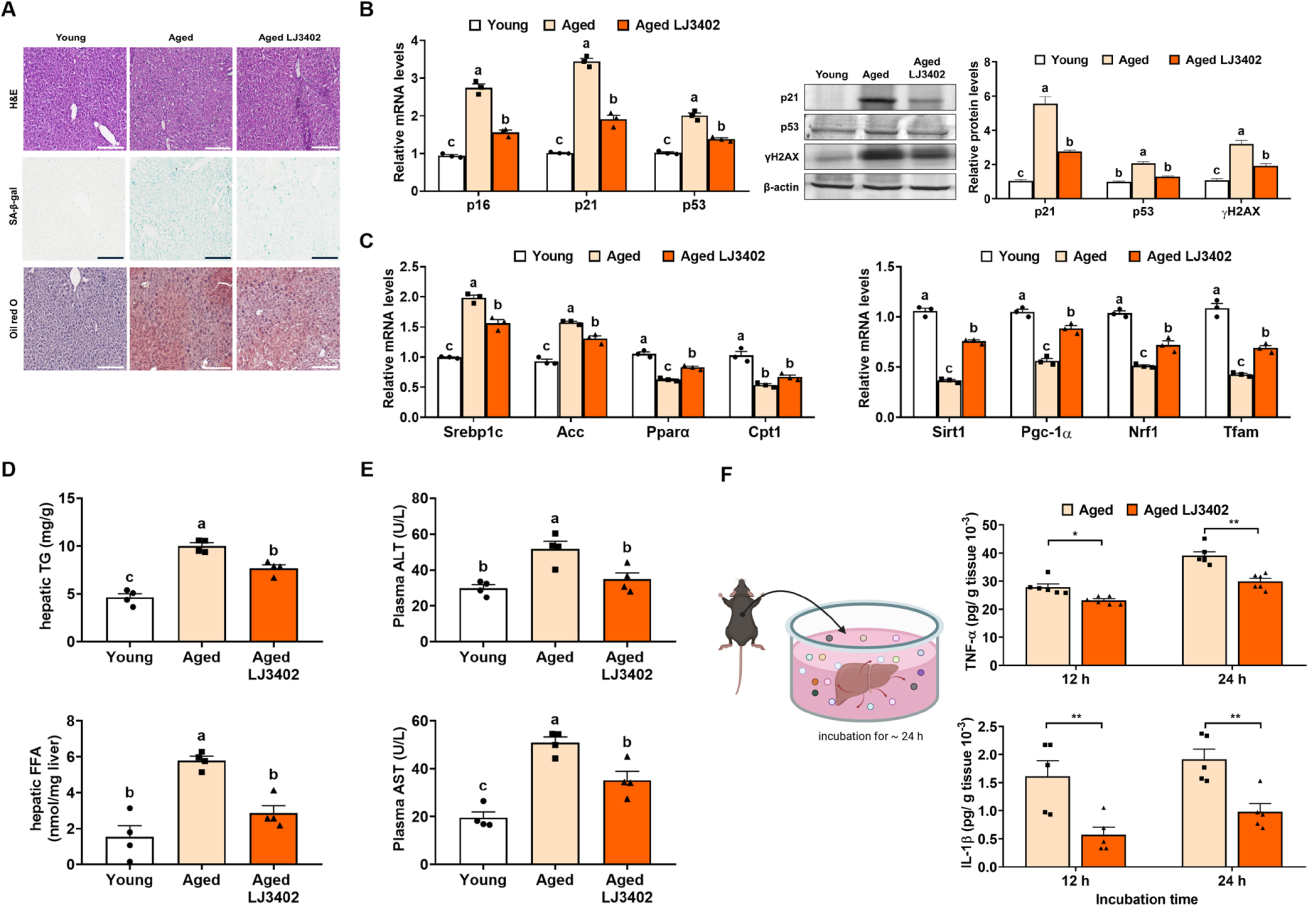
LJ3402 prevents hepatic senescence and mitochondrial dysfunction in aged mice. (A) Representative images of hematoxylin and eosin (H&E) (top), SA‐β‐gal (middle), and Oil Red O (bottom) staining on frozen liver sections from the indicated group. (B) The mRNA levels of marker genes involved in senescence (*p16*, *p21*, *p53*) and protein levels (γH2AX, p21, p53) in the liver of young and aged mice were determined by RT‐qPCR or immunoblotting (*n* = 3; three randomly selected samples per group). (C) Expression of marker genes involved in lipid metabolism (*Srebp1c*, *Pparα*, *Cpt1*) and mitochondrial homeostasis (*Sirt1*, *Pgc‐1α*, *NRF1*, *TFAM*) in the liver was determined using RT‐qPCR. (D) Hepatic triglyceride (TG), total cholesterol (TC), and free fatty acid (FFA), and (E) plasma levels of alanine aminotransferase (ALT) and aspartate aminotransferase (AST) (*n* = 5; three randomly selected samples per group). (F) Livers separated from mice were incubated in culture medium, and the media were collected after 12 h and 24 h of incubation; the concentrations of TNF‐α and IL‐1β in the media were measured (*n =* 5; three randomly selected samples per group). All data are presented as the mean ± SEM. * *p* < 0.05 and **p < 0.01. Different lowercase letters above the bars indicate statistically significant differences (*p* < 0.05).

Because the liver orchestrates systemic metabolism, age‐associated declines in hepatic function contribute to metabolic disease. In histological analyses, aged‐LJ3402 mice showed reduced hepatic lipid accumulation and less senescence‐associated β‐galactosidase (SA‐β‐gal) staining than aged‐control mice (Figure 2A). Consistently, aged‐control mice had higher hepatic mRNA levels of senescence‐related genes (*p16*, *p21*, and *p53*) than young mice, whereas administration of LJ3402 reduced these transcripts by 32%–45% (*p* < 0.05; Figure 2B). Immunoblotting further showed that LJ3402 administration reduced p21, p53, and γH2AX protein levels in aged liver tissue by 51%, 38%, and 40%, respectively, compared with aged‐control mice (*p* < 0.05), suggesting reduced hepatic senescence. Moreover, relative to young mice, aged‐control mice displayed increased hepatic expression of lipogenic genes (*Srebp1c* and *Acc*) and decreased expression of genes involved in mitochondrial homeostasis (*Sirt1*, *Pgc‐1α*, *NRF1*, *TFAM*) and fatty acid oxidation (*Cpt1* and *Pparα*) (*p* < 0.05). LJ3402 administration significantly blunted these age‐related expression changes (*p* < 0.05; Figure 2C). We next analyzed the effect of LJ3402 on hepatic lipid profile. As shown in Figure 2D, TG and FFA levels were significantly higher in aged‐control mice than in young mice (*p* < 0.05). However, administration of LJ3402 reduced hepatic TG and FFA levels in aged mice by 24% and 51%, respectively (*p* < 0.05). Furthermore, plasma ALT and AST—indicators of liver injury—were 1.7‐fold and 2.5‐fold higher, respectively, in aged‐control mice than in young mice (*p* < 0.05; Figure 2E). However, these plasma enzymes were reduced by 31%–32% in aged‐LJ3402 mice (*p* < 0.05). Furthermore, when ex vivo liver explants from each group of aged mice were incubated in the culture media for 12 or 24 h, the TNFα and IL‐1β in conditioned media (CM) from aged‐LJ3402 mice were 20%–27% and 49%–65%, respectively, lower than CM from aged‐control livers (*p* < 0.05; Figure 2F). These findings suggest that LJ3402 may attenuate age‐associated hepatocyte senescence and liver dysfunction.

### 3.3 LJ3402 Enhances *Sirt1* Transcription in AML12 Hepatocytes Through the PGC‐1α–PPARα Pathway

**FIGURE 3:**
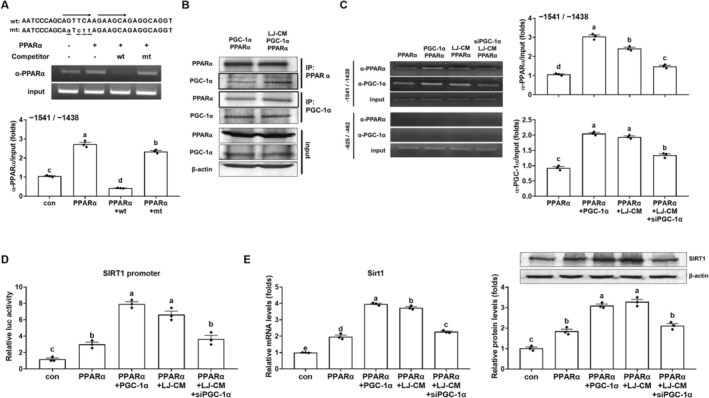
LJ3402‐mediated enhancement of hepatic SIRT1 expression through the PGC‐1α–PPARα pathway. (A) A ChIP analysis in AML12 hepatocytes using an anti‐PPARα. AML12 cells were transfected with PPARα expression plasmids, and approximately 20 μg excess of wild type (wt) or mutated (mt) oligonucleotides were added as competitors. The *Sirt1* promoter region containing a PPRE (−1541 to −1438 bp) were amplified from the immunoprecipitated DNA using PCR (upper) and qPCR (lower). (B) IP analysis of the interaction between PPARα and PGC‐1α in AML12 hepatocytes using the indicated antibodies. (C and D) AML12 cells were transfected with the indicated expression plasmids and *PGC‐1α* siRNA in the presence or absence of LJ3402‐CM (LJ‐CM) for 24 h. (C) A ChIP analysis in AML12 hepatocytes using an anti‐PPARα and PGC‐1α antibody, respectively. The *Sirt1* promoter region containing a PPRE (−1541 to −1438 bp) and the region lacking a PPRE (−625 to −482 bp) were amplified from the immunoprecipitated DNA using PCR (left) and qPCR (right). (D) Luciferase activity analysis in HEK293T cells. After 12 h of transfection of a reporter plasmid (*Sirt1* promoter‐luc) along with the indicated expression plasmids and siRNA, cells were treated with LJ‐CM for 24 h. (E) The mRNA and protein levels of SIRT1 in AML12 hepatocytes were determined by RT‐qPCR and immunoblotting respectively. All data are presented as the mean ± SEM. Different lowercase letters above the bars indicate statistically significant differences (*p* < 0.05).

SIRT1 promotes mitochondrial function by upregulating PGC‐1α, but whether PGC‐1α regulates SIRT1 expression is unclear. Sequence analysis identified a direct repeat 1 site—a putative PPARα response element (PPRE)—in the mouse *Sirt1* promoter at −1497 to −1485 bp. Because PGC‐1α functions as a coactivator of PPARα, we first tested PPARα occupancy at this PPRE by a ChIP assay. ChIP analysis showed that PPARα overexpression increased PPARα recruitment to the Sirt1 promoter region (−1541 to −1438 bp) encompassing the PPRE (*p* < 0.05; Figure 3A). Notably, this PPARα binding was abolished by an excess of the wild‐type PPRE but not by a mutated PPRE (*p* < 0.05). Co‐IP with anti‐PPARα and anti‐PGC‐1α antibodies showed that LJ3402‐CM increased the PGC‐1α–PPARα interaction, compared with vehicle treatment (Figure 3B). Consistently, ChIP assays in AML12 hepatocytes overexpressing PPARα showed that PGC‐1α and LJ3402‐CM increased the binding of PPARα and PGC‐1α to the *Sirt1* promoter region containing the PPRE by 2.2–2.8‐fold and 2.0–2.2‐fold, respectively, compared with PPARα alone (*p* < 0.05; Figure 3C), but not at a downstream region lacking a PPRE (−627 to −428 bp). In contrast, *PGC‐1α* siRNA markedly reduced the LJ3402‐enhanced occupancy of both factors at this promoter region (*p* < 0.05). To test whether LJ3402 augments PPARα‐driven *Sirt1* promoter activity via PGC‐1α, we performed luciferase assays in HEK293T cells using a human *SIRT1* promoter reporter containing a PPRE. Both PGC‐1α and LJ3402‐CM further enhanced PPARα‐dependent *SIRT1* promoter activity compared with PPARα alone (*p* < 0.05; Figure 3D). However, co‐administration of *PGC‐1α* siRNA with LJ3402‐CM blunted the LJ3402‐CM enhancement of PPARα transcriptional activity (*p* < 0.05). RT‐qPCR and immunoblotting analyses showed that both PGC‐1α and LJ3402‐CM increased PPARα‐induced SIRT1 expression in AML12 hepatocytes compared with PPARα alone. This enhancing effect of LJ3402 on SIRT1 expression was abolished by PGC‐1α knockdown (*p* < 0.05; Figure 3E).

### 3.4 LJ3402 Enhances Hepatic Mitochondrial Function Through a Positive Regulatory Loop Between PGC‐1α and SIRT1

**FIGURE 4:**
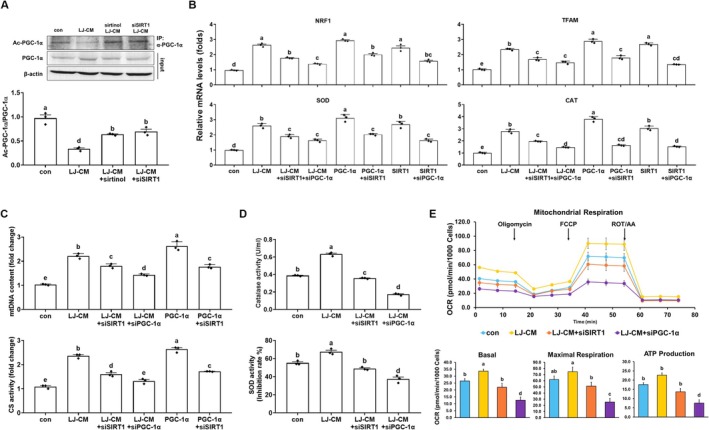
LJ3402 improves mitochondrial function in AML12 hepatocytes through a positive regulatory loop between PGC‐1α and SIRT1. (A) Acetylation of PGC‐1α in AML12 hepatocytes. (A‐E) AML12 hepatocytes were transfected with the indicated expression plasmids and siRNAs for 24 h. Cells were then incubated with LJ3402‐CM (LJ‐CM) and 50 μM sirtinol for an additional 24 h, as indicated. (B) The mRNA levels of genes regulated by PGC‐1α, which are essential for PGC‐1α function, were analyzed using RT‐qPCR. (C) The mtDNA copy number and citrate synthase (CS) activity in AML12 hepatocytes. (D) The activities of catalase (CAT) and superoxide dismutase (SOD) in AML12 hepatocytes, as indicated. (E) The oxygen consumption rates (OCRs) in AML12 hepatocytes treated with indicated siRNAs and LJ‐CM were measured in the basal condition and in the presence of 2.5 μM oligomycin, 1 μM FCCP, and 1 μM rotenone/antimycin A at the indicated time point. All data are presented as the mean ± SEM. Different lowercase letters above the bars indicate statistically significant differences (*p* < 0.05).

To determine whether LJ3402 induces SIRT1‐dependent deacetylation of PGC‐1α, PGC‐1α was immunoprecipitated from AML12 hepatocytes with an anti‐PGC‐1α antibody, and acetylation of immunoprecipitated PGC‐1α was assessed with an anti–acetyl‐lysine antibody. In the immunoblotting analysis, LJ3402‐CM reduced PGC‐1α acetylation in AML12 hepatocytes by 60% compared with untreated cells (*p* < 0.05; Figure 4A). However, the addition of 50 μM sirtinol or *SIRT1* siRNA partially prevented LJ3402‐induced PGC‐1α deacetylation (*p* < 0.05), supporting a positive feedback loop between SIRT1 and PGC‐1α. We next investigated whether SIRT1 is required for LJ3402 enhancement of PGC‐1α‐regulated gene expression in AML12 hepatocytes. In RT‐qPCR analyses, LJ3402 and SIRT1 each increased mRNA levels of genes associated with mitochondrial biogenesis (*NRF1* and *TFAM*) and antioxidation (*SOD1* and *CAT*) by 2.3–3‐fold compared to control AML12 cells, similar to PGC‐1α overexpression (*p* < 0.05; Figure 4B). In addition, silencing SIRT1 attenuated the effect of PGC‐1α overexpression, and silencing PGC‐1α attenuated the effect of SIRT1 overexpression on these mitochondrial genes (*p* < 0.05). Likewise, silencing SIRT1 or PGC‐1α in LJ3402‐CM–treated AML12 cells reduced LJ3402‐induced expression of these genes (*p* < 0.05), further confirming that a positive feedback pathway between PGC‐1α and SIRT1 mediates LJ3402‐induced mitochondrial gene expression. LJ3402 increased mtDNA copy number and CS activity in AML12 hepatocytes (*p* < 0.05; Figure 4C). However, these promoting effects were lost when either PGC‐1α or SIRT1 was silenced (*p* < 0.05). Similarly, SIRT1 silencing abolished the PGC‐1α‐induced increases in mtDNA copy number and CS activity (*p* < 0.05). We then examined the role of the PGC‐1α–SIRT1 axis in LJ3402 effects on antioxidant function. LJ3402‐CM increased CAT and SOD activities by 1.6‐fold and 1.2‐fold, respectively, compared with control AML12 cells (*p* < 0.05; Figure 4D). However, silencing SIRT1 or PGC‐1α eliminated the LJ3402‐CM–mediated increases in the activities of these antioxidant enzymes (*p* < 0.05). In addition, LJ3402‐CM significantly increased basal, maximal, and ATP production OCRs by 1.2–1.29‐fold in AML12 hepatocytes (*p* < 0.05; Figure 4E). These LJ3402 effects were abolished by silencing SIRT1 or PGC‐1α, with a stronger inhibition after PGC‐1α silencing (*p* < 0.05). Together, these results indicate that the PGC‐1α–SIRT1 axis is critical for LJ3402‐mediated improvements in hepatic mitochondrial function.

### 3.5 LJ3402 Reduces H_2_O_2_ ‐Induced Senescence of AML12 Hepatocytes Through Stimulation of SIRT1‐Mediated Deacetylation of p53

**FIGURE 5:**
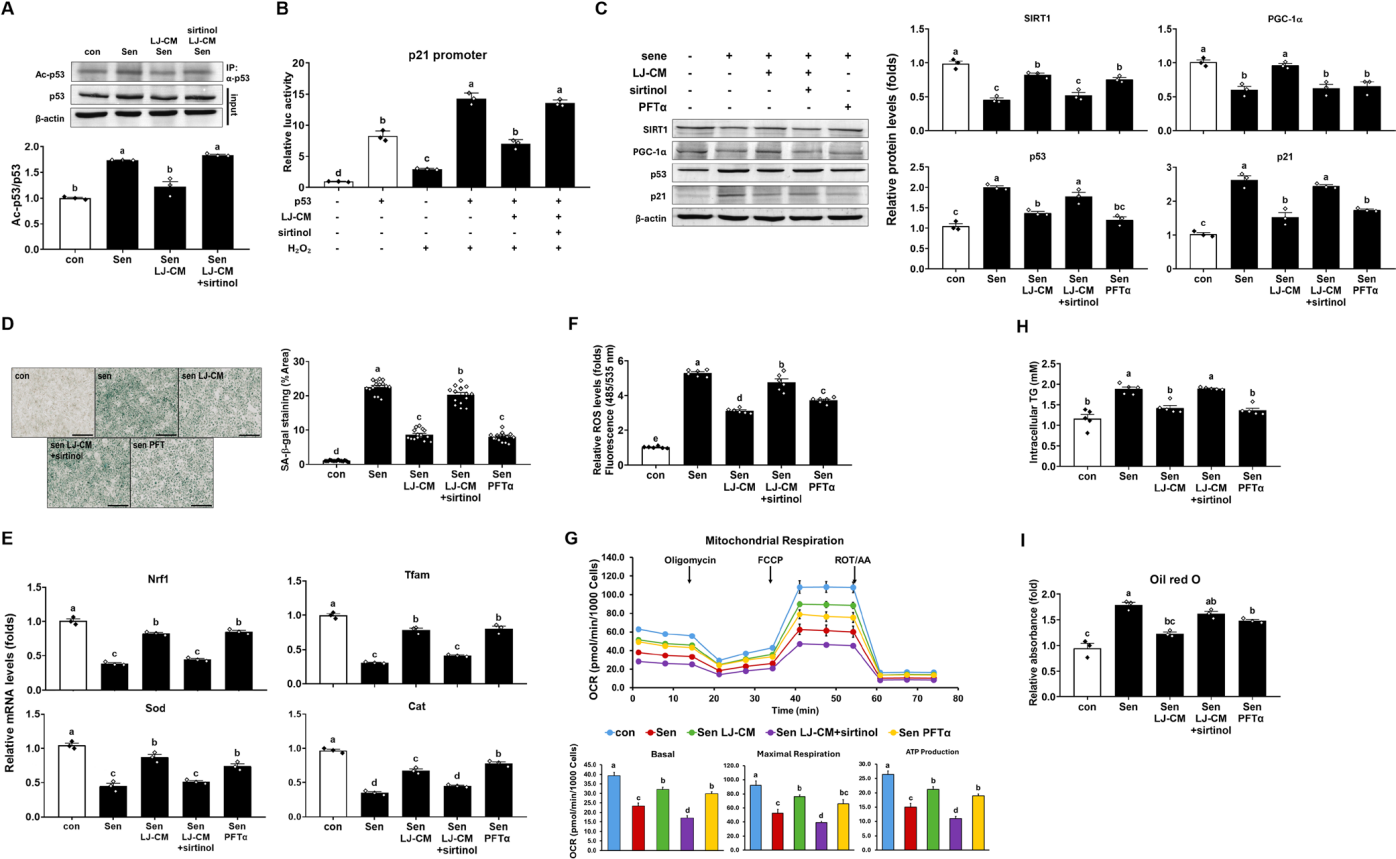
SIRT1 modulates LJ3402‐mediated suppression of p53 function in senescent AML12 hepatocytes. (A) Acetylation of p53 after 5 days of treatment with LJ3402‐CM (LJ‐CM) and 50 μM sirtinol during H_2_O_2_‐induced senescence. (A, C–I) Senescence of AML12 hepatocytes was induced by 1 mM or 750 μM H_2_O_2_ for 1 h per day for 7 days. LJ‐CM, 50 μM sirtinol, or 20 μM PFTα were added on days 3–7 during H_2_O_2_ exposure. (B) Luciferase reporter activity in HEK293T cells transfected with a reporter plasmid (*p21* promoter–luc) and a p53 expression plasmid. Twelve hours after transfection, cells were treated with LJ‐CM and 50 μM sirtinol for 24 h with or without 100 μM H_2_O_2_. (C) The mRNA and protein levels of p21, p53, PGC‐1α, and SIRT1 measured by RT‐qPCR and immunoblotting, respectively. (D) SA‐β‐gal staining. (E) mRNA levels of genes involved in mitochondrial biogenesis (*NRF1* and *TFAM*) and oxidant defense (*CAT* and *SOD*). (F) ROS levels. (G) Oxygen consumption rates (OCRs) measured at baseline and after treatment with 2.5 μM oligomycin, 1 μM FCCP, or 1 μM rotenone/antimycin A. (H) Oil Red O staining. (I) Intracellular TG levels in senescent AML12 hepatocytes treated with LJ‐CM, sirtinol, and PFTα. All data are presented as the mean ± SEM. Different lowercase letters above the bars indicate statistically significant differences (*p* < 0.05).

We next tested whether LJ3402 alters p53 acetylation in senescent AML12 hepatocytes, given that SIRT1 deacetylates p53. Senescence of AML12 cells was induced with H_2_O_2_ [25]. p53 was immunoprecipitated from senescent AML12 cells using an anti‐p53 antibody and its acetylation was assessed using an anti–acetyl‐lysine antibody. H_2_O_2_‐induced senescence increased p53 acetylation in AML12 cells by 1.7‐fold compared with vehicle (*p* < 0.05; Figure 5A). LJ3402‐CM reduced p53 acetylation in these senescent cells by 30% compared with H_2_O_2_ alone (*p* < 0.05), and 50 μM sirtinol abolished this LJ3402‐CM effect (*p* < 0.05). To test whether LJ3402 modulates p53 activity through SIRT1, we used a p21 promoter–luciferase reporter containing a p53 response element in HEK293T cells. p53 increased p21 promoter activity, and H_2_O_2_ further enhanced this p53 transactivation (*p* < 0.05; Figure 5B). LJ3402‐CM abolished the H_2_O_2_‐induced increase in p53 transcriptional activity, whereas sirtinol markedly reduced this LJ3402‐CM effect on p53 activity (*p* < 0.05). Consistently, immunoblotting analyses showed that H_2_O_2_‐induced senescence increased p53 and p21 and reduced PGC‐1α and SIRT1 protein levels in AML12 hepatocytes (*p* < 0.05; Figure 5C). However, these H_2_O_2_ effects were reversed by LJ3402‐CM or pifithrin‐α (PFTα), a p53‐specific inhibitor. In addition, co‐treatment of sirtinol with LJ3402‐CM abrogated the LJ3402‐mediated normalization of these senescence‐induced changes in gene expression (*p* < 0.05). Consistently, H_2_O_2_ increased SA‐β‐gal staining in AML12 hepatocytes compared with control cells, whereas LJ3402‐CM or PFTα reduced this H_2_O_2_‐induced staining by 62% and 65%, respectively, relative to H_2_O_2_ treatment alone (*p* < 0.05; Figure 5D). In addition, co‐treatment of sirtinol with LJ3402‐CM restored SA‐β‐gal staining in senescent AML12 hepatocytes (*p* < 0.05). Accumulating evidence indicates that under stress, p53 promotes mitochondrial dysfunction by downregulating PGC‐1α [16, 26]. We therefore tested whether LJ3402 reverses H_2_O_2_‐mediated suppression of PGC‐1α–regulated mitochondrial genes. RT‐qPCR analysis showed that H_2_O_2_ reduced *NRF1*, *TFAM*, *SOD*, and *CAT* mRNA by 51%–70% relative to control AML12 cells, whereas LJ3402 and PFTα mitigated the effect of H_2_O_2_ on their expression (*p* < 0.05; Figure 5E). Consistently, sirtinol abolished the LJ3402‐mediated protection against H_2_O_2_‐induced reductions in these mitochondrial genes (*p* < 0.05). Because mitochondrial dysfunction elevates ROS, we quantified ROS in senescent AML12 hepatocytes. H_2_O_2_ increased ROS by 5.1‐fold relative to untreated cells (*p* < 0.05; Figure 5F). LJ3402‐CM or PFTα decreased ROS by 42% and 30%, respectively, in H_2_O_2_‐treated cells (*p* < 0.05), whereas sirtinol abolished the LJ3402‐CM effect on ROS in these senescent hepatocytes (*p* < 0.05). Both PFTα and LJ3402‐CM increased basal and maximal OCRs and ATP production in H_2_O_2_‐treated AML12 hepatocytes compared with H_2_O_2_ alone (*p* < 0.05; Figure 5G). However, this suppressive effect of LJ3402‐CM on the H_2_O_2−_induced reduction in OCRs was markedly decreased by sirtinol (*p* < 0.05). We next evaluated LJ3402 effects on lipid accumulation in H_2_O_2_‐treated AML12 hepatocytes. Senescent AML12 cells showed increased cellular TG and Oil Red O staining, compared with control cells (*p* < 0.05; Figure 5H,I). However, LJ3402‐CM and PFTα reduced TG levels and Oil Red O staining in senescent AML12 hepatocytes, whereas sirtinol reversed the LJ3402‐CM effect (*p* < 0.05). These data indicate that LJ3402 reduces senescence‐induced hepatic lipid accumulation in part by promoting SIRT1‐dependent suppression of p53 function.

## 4 Discussion

The liver is a central metabolic organ essential for physiological homeostasis, including energy metabolism. Liver function, however, progressively declines with age, contributing to age‐related hepatic disorders such as MASLD [27]. This study demonstrated that LJ3402 increases SIRT1 expression by inducing PGC‐1α–mediated coactivation of PPARα. Furthermore, SIRT1 deacetylates PGC‐1α and p53, thereby mitigating mitochondrial dysfunction and hepatocyte senescence and, consequently, attenuating the progression of age‐related fatty liver.

Declining SIRT1 expression in hepatocytes is frequently associated with mitochondrial dysfunction and chronic liver disease [28, 29]. Its role in hepatic mitochondrial function has been thought to occur primarily through regulation of PGC‐1α, a master regulator of mitochondrial biogenesis and function [10, 30]. However, a role for PGC‐1α in regulating SIRT1‐mediated mitochondrial homeostasis had not been demonstrated. Our findings revealed that LJ3402 enhances SIRT1 expression by promoting PGC‐1α recruitment to the Sirt1 promoter region containing a PPRE, thereby strengthening PPARα transcriptional activity. This effect of LJ3402 on Sirt1 transcription was further supported by LJ3402‐induced PGC‐1α deacetylation, which was abrogated by sirtinol, a SIRT1 inhibitor, demonstrating that LJ3402 improves mitochondrial quality by establishing a positive regulatory loop between PGC‐1α and SIRT1. Consistent with this, LJ3402 increased mtDNA copy number and OCRs in hepatocytes. It also prevented age‐induced alterations in mitochondrial gene‐expression programs, limited hepatic ROS, and lowered plasma ALT and AST, thereby attenuating age‐related MASLD development and progression.

Mitochondrial dysfunction, a well‐established driver of cellular senescence, is closely associated with p53‐dependent cellular senescence [26]. Notably, hepatic expression of p21, a downstream p53 target gene, increases in parallel with MASLD progression [31, 32]. This study showed that LJ3402‐induced SIRT1 suppresses p53 activity by promoting deacetylation, thereby reducing hepatocyte senescence, as indicated by lower expression of senescence markers, decreased hepatic SA‐β‐gal activity and γH2AX, and reduced circulating SASP factors. Age‐related hepatocyte senescence significantly alters liver function by releasing SASPs that remodel the hepatic microenvironment, propagate senescence to neighboring cells, exacerbate inflammation, and promote MASLD development and progression [33]. Thus, LJ3402 may limit the spread of senescent cells in the liver by modulating SASP secretion.

Probiotics, including *Lactobacillus* sp., have been linked to healthy aging [34, 35, 36]. Although probiotics have various beneficial effects on health, including alleviation of oxidative stress, chronic inflammation, and metabolic dysfunction, the mechanisms by which they ameliorate age‐related liver dysfunction remain unclear. Here, age‐related hepatic senescence and dysfunction were correlated positively with p53 activation in hepatocytes and inversely with hepatic PGC‐1α and SIRT1. LJ3402 administration restored these age‐associated hepatic changes, including gene‐expression profiles, via SIRT1‐mediated suppression of p53, implicating the PGC‐1α–SIRT1–p53 axis as critical for LJ3402's hepatoprotective effect. Moreover, the dose used here corresponds to approximately 3.25 × 10^9^ CFU/day in humans, within the range of probiotic health supplements, suggesting a potential approach to treat age‐related metabolic disorders.

Aging is strongly associated with metabolic abnormalities, including insulin resistance and reduced energy expenditure [3, 37, 38, 39]. Hepatocyte senescence contributes to these abnormalities through SASP secretion [32, 33]. Consistent with LJ3402's suppression of circulating inflammatory cytokines (TNFα and IL‐1β), LJ3402 administration mitigated age‐related insulin resistance and impaired thermoregulation, as demonstrated by improved glucose and insulin tolerance and increased cold resistance.

Our study also has limitations. First, although LJ3402 activates the PGC‐1α–SIRT1–p53 pathway to attenuate age‐associated liver dysfunction by improving mitochondrial function, both PGC‐1α and SIRT1 also regulate antioxidant defenses and mitochondrial homeostasis through interactions with transcription factors such as Nrf2 [19]. Thus, the PGC‐1α–SIRT1 axis may mediate additional protective effects via other transcriptional programs, including Nrf2 signaling. Further studies dissecting these networks are warranted. Second, although we detected a statistically significant protective effect of LJ3402 on age‐related liver dysfunction in male mice, our sample size was modest, which may have limited our ability to detect age‐related changes in body weight. Third, because MASLD is more prevalent in males, we used only male mice to model age‐related liver dysfunction and to maintain consistency with our previous diet‐induced obesity model. Future studies with larger cohorts, including females, are needed to define LJ3402's benefits across sexes in age‐related metabolic disorders. Together, our findings demonstrate that LJ3402 preserves liver function during aging by improving mitochondrial function and suppressing cellular senescence through the PGC‐1α–SIRT1–p53 pathway, supporting LJ3402 as a potential preventive strategy for age‐related metabolic disorders.

## Author Contributions

**Eunjeong Hong:** conceptualization, investigation, data curation, writing – original draft. **Garam Yang:** formal analysis, investigation. **Sejong Oh:** resources. **Eungseok Kim:** conceptualization, supervision, writing – review and editing.

## Funding

This work was supported by the Ministry of Science and ICT, South Korea, NRF‐2021R1A2C1005894.

## Ethics Statement

All animal experiments were approved by the Institutional Animal Care and Use Committee at Chonnam National University (CNU IACUC‐YB‐2020‐86, Approved date: 7 October 2020).

## Conflicts of Interest

The authors declare no conflicts of interest.

## Data Availability

The data that support the findings of this study are available from the corresponding author upon reasonable request.
